# Carbon Footprint of the Pork Product Chain and Recent Advancements in Mitigation Strategies

**DOI:** 10.3390/foods12234203

**Published:** 2023-11-22

**Authors:** Pan Yang, Miao Yu, Xianyong Ma, Dun Deng

**Affiliations:** Key Laboratory of Animal Nutrition and Feed of South China, Ministry of Agriculture and Rural Affairs, Institute of Animal Science, Guangdong Academy of Agricultural Sciences, Guangzhou 510640, China; yangpan@gdaas.cn (P.Y.); yumiao@gdaas.cn (M.Y.); maxianyong@gdaas.cn (X.M.)

**Keywords:** carbon footprint, mitigation strategy, greenhouse gas emission, pork production chain

## Abstract

The carbon footprint of pork production is a pressing concern due to the industry’s significant greenhouse gas emissions. It is crucial to achieve low-carbon development and carbon neutrality in pork production. Thus, this paper reviewed the recent studies about various sources of carbon emissions throughout the current pork production chain; feed production, processing, and manure management are the major sources of carbon emissions. The carbon footprint of the pork production chain varies from 0.6 to 6.75 kg CO_2_e·kg^−1^ pig live weight, and the carbon footprint of 1 kg of pork cuts is equivalent to 2.25 to 4.52 kg CO_2_e. A large reduction in carbon emissions could be achieved simultaneously if combining strategies of reducing transportation distances, optimizing farmland management, minimizing chemical fertilizer usage, promoting organic farming, increasing renewable energy adoption, and improving production efficiency. In summary, these mitigation strategies could effectively decrease carbon emissions by 6.5% to 50% in each sector. Therefore, a proper combination of mitigation strategies is essential to alleviate greenhouse gas emissions without sacrificing pork supply.

## 1. Introduction

Global climate change has become a pressing issue, particularly in reducing anthropogenic carbon dioxide (CO_2_) emissions. Reducing greenhouse gas (GHG) emissions is one of the effective approaches to addressing global warming. According to the latest report by the United Nations Environment Programme (UNEP), global average GHG emissions between 2010 and 2019 were approximately 54.4 petagrams of CO_2_ equivalent (PgCO_2_e), reaching a peak of 56.4 PgCO_2_e in 2019 [1]. Agriculture is a large contributor to GHG emissions; the top ten emitters are China, the United States of America, India, the Russian Federation, Indonesia, Brazil, Japan, Iran, Canada, and Mexico [2]. The Food and Agriculture Organization of the United Nations (FAO) reported that livestock production contributes 10% to 12% of total global GHG emissions. Cattle, pigs, and poultry collectively represent the top three sources of carbon emissions among various animal species, with pork production being the second contributor. Pork production accounts for approximately 14% in the agricultural sector [3]. According to GHG emissions data from the pig production chain reported by the FAO (Table 1 and Table 2), feed production accounts for 42.64% of total emissions, totaling 3.6 teragrams (Tg) of CO_2_ equivalent annually. Manure management is the second largest contributor in whole industry, accounting for 39.15% of emissions, totaling 3.31 Tg of CO_2_ equivalent annually. The focus of transforming livestock farming toward a low-carbon model is centered on reducing carbon emissions and achieving carbon neutrality in the pork industry. It is crucial to conduct comprehensive analyses of the carbon footprint associated with pork production for establishing sustainable production systems and achieving the national carbon neutrality objective. Thus, this article offers a comprehensive overview of recent research advancements in understanding the carbon footprint of the pork production chain and provides specific insights and references for promoting the adoption of a low-carbon meat production chain.

## 2. System Boundary of Pork Products

**Figure 1:**
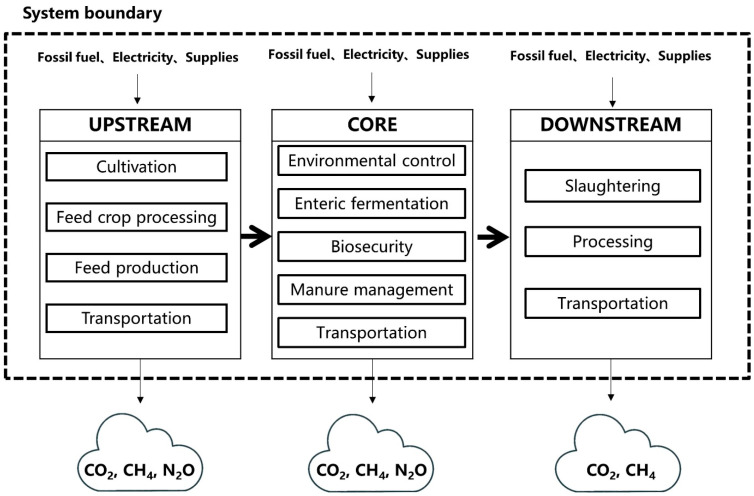
Life cycle system boundary of the pork production chain.

A Life Cycle Assessment (LCA) method was used to analyze the carbon emissions of the pork products in this study. The system boundary is shown in Figure 1. The cradle-to-gate process consists of three main components, namely the upstream, the core, and the downstream. The upstream comprises cultivation, feed crop processing, feed production, and feed transportation; the core production process includes environmental control, enteric fermentation, manure management, biosecurity, and pig transportation; and the downstream component refers to slaughter, pork processing, and transportation.

## 3. Source of GHG Emissions

### 3.1. Transportation

Transportation during the whole pork production chain typically involves three stages. Firstly, feed ingredients are transported from their point of origin regions to the feed mills. Secondly, complete feed is transported from the feed mills to the farms. Lastly, pigs are transported from the farms to the slaughterhouses. Studies have reported that the transportation of feed ingredients in pork production systems is a significant source of GHG emissions in feed production, with feed processing and transportation accounting for 34% of the carbon footprint in feed production [4]. The transportation of animals to the slaughterhouse and the delivery of processed products to markets is another source contributing to carbon emissions. Chen et al. [5] calculated the carbon emissions in the pork supply chain in China, with the transportation segment accounting for 52.37% of the GHGs emitted over the entire supply chain. Furthermore, the contribution of carbon emissions for transportation in the pork supply chain varies from 2.2% to 10% depending on regional conditions and practices (Table 3).

### 3.2. Feed Production

The carbon footprint of feed production is caused by the energy consumption from the cultivation of crops to feed processing. The carbon emissions associated with feed crop cultivation mainly include fossil fuel consumption involved with seeds, pesticides, and fertilizers, as well as agricultural machinery. According to Zhou et al. [4], carbon emissions during the feed crop cultivation stage account for 66% of total emissions in the entire feed production. The energy consumption in feed processing includes the following main components: the electricity consumption during the process includes the power required for material reception via conveyor, elevator, cleaning sieve, and magnetic separator, as well as the power consumption during bagged raw material transportation via conveyor belts and mechanical stacking. Additionally, energy is consumed by the elevators and crushers during the grinding process, and power is used for material transportation and mixing using scrapers and mixers during the mixing process. Furthermore, electricity and steam are utilized during the stages of feed conditioning and pelleting, and finally, electricity is consumed during cooling, storage, and packaging of finished products [10]. Electricity consumption indirectly leads to CO_2_ emissions, and the emission coefficient depends on factors such as fuel type, electricity sources, and electricity production efficiency. Fang et al. [11] reported that processing 1000 kg of pig feed generates 28.03 kg of CO_2_. Based on the reported 322 Tg of pig feed per year in the world [12], the CO_2_ emissions from feed processing alone for the entire year amounted to approximately 9.03 Tg, indicating significant potential for carbon mitigation in feed production and processing. However, there is limited research data on the energy consumption of different feed processing stages, and these data may vary due to different experimental conditions and production management systems. Based on current research findings, feed production and processing require a substantial amount of energy input, which is largely dependent on fossil energy sources and constitutes a major portion of the total energy consumption in pork production. Feed production and processing constitute a substantial portion of the total energy consumption in the pork production chain, surpassing the 50% reported by previous study [13]. In summary, the contribution of feed production ranges from 26% to 68% (Table 3).

### 3.3. Pig Production

The modern pig industry exhibits characteristics of intensive and automated production. Automatic feeding management and environmental control include a feed transport system, automatic feeding systems, ventilation, insulation, and heating in pig houses. However, the environmental control equipment consumes much electricity during the production process [14]. Apart from the daily lighting systems in the pig houses, 16 h of lighting are provided for sows to promote animal estrus. Although the proportion of electricity consumed by lighting is relatively low compared to the total electricity consumption, this energy consumption should not be ignored when calculating the carbon emissions of the entire pig production chain. Paris et al. [15] summarized the research progress on energy consumption in livestock production and pointed out that 50% of the energy required to produce 1 kg of pork is related to electricity consumption on pig farms.

Various consumables are required in the pig production process, including medications, cleaning supplies, personal protective equipment, maintenance, vaccinations, and semen collection supplies. Animal medications mainly include vaccines, immune enhancers, and treatment drugs used in the immunization processes; the production and transportation processes of medications consume energy and contribute to GHG emissions. Maintaining cleanliness and hygiene in pig pens requires the use of various cleaning supplies such as disinfectants and detergents; the production and transportation processes of these cleaning supplies also consume energy and result in carbon emissions. Additionally, the maintenance of pens and electrical equipment during the operation of pig farms accounts for energy use, and the production and transportation of these maintenance items also consume energy and contribute to carbon footprints; these footprints can indeed vary significantly due to regional differences, surrounding environmental conditions, and management practices, making them more challenging to quantify. Although comprehensive life cycle research data on consumables in pig production is currently lacking, it should be noted that factors such as different regions, scales, and management levels can affect carbon emissions during the pork supply chain. According to Table 3, the contribution of the pig production sector ranges from 2.6% to 17.0%.

### 3.4. Respiration and Gut Fermentation

Animal respiration is one of source for CO_2_ production. Under aerobic conditions, organic matter is metabolized through enzymatic reactions to generate energy for maintaining biological functions. Since the CO_2_ emissions from respiration is part of the Earth ecosystem, these are not considered in carbon footprint accounting. The FAO does not include CO_2_ produced by animal respiration in its Global Livestock Environmental Assessment Model (GLEAM 3.0). However, Cai et al. [16] reported that respiratory emissions account for approximately 5% to 10% of the annual CO_2_ emissions from fossil fuel combustion, highlighting the importance of considering CO_2_ from respiration in ecosystem carbon emissions and GHG management. It is necessary to research whether the CO_2_ generated by animal respiration can be absorbed and mitigated by ecosystems, especially considering the current large population of animals.

A previous study was carried out to estimate CO_2_ exhalation, and the pig model was proposed to express the CO_2_ exhalation function of body weight (BW) in the range from 20 to 120 kg BW [17]:

CO_2_ exhalation (kg/day) = 0.136 BW^0.573^ (1)

However, a recent study carried out to estimate and predict CO_2_ emission rate as a function of the mass of pigs and feed consumption recommended employing an exponential-based regression model by taking feed intake as an input variable in predicting the daily CO_2_ emission rate per pig [18]. Thus, more studies should be undertaken to estimate CO_2_ exhalation and provide effective information regarding a carbon emission inventory of pig production.

Pig intestines harbor a variety of microorganisms, including methane-producing bacteria [19]. During the digestive process, undigested carbohydrates undergo fermentation in the hindgut, producing short-chain fatty acids and gases such as CH_4_, CO_2_, and H_2_ [20]. Methane-producing bacteria in the intestines convert CO_2_ and H_2_ into CH_4_. These gases are released into the atmosphere through the mouth and anus, contributing to global warming. Li et al. [6] reported that GHG emissions from pig intestinal fermentation accounted for 9.6% of the emissions from large-scale pig farming. The CH_4_ emissions for pigs are calculated according to the equations by Kuhla and Viereck (CH_4_ production per head and day = 0.07 × BW^0.99^) [21]. According to the latest data, the global pig production for market was 975 million heads [2]. Due to the large population of pigs in the world, the CH_4_ emissions from pig intestinal fermentation constitute an important source of GHG emissions in agricultural production. According to the Tier 1 methodology from the Intergovernmental Panel on Climate Change (IPCC) guidelines for national inventories [22], enteric CH_4_ is estimated at 1.5 or 1.0 kg per head per year in developed or developing countries, corresponding to 4.1 or 2.7 g CH_4_ per day, whatever the diet composition and physiological stage. According to the guidelines [23], CH_4_ emissions from enteric production of growing or adult pigs can be estimated using following equations:

Growing pigs: Enteric CH_4_ = 6.7 × (0.5 × Res) (2)

Adult pigs: Enteric CH_4_ = 13.4 × (0.6 × Res) (3)

where residue (Res) may be estimated as follows:

Res = 100 − Ash − Protein − Fat − Starch − Sugars (or default value of 2% dry matter)(4)

All the feed components are expressed as % dry matter. Furthermore, Misiukiewicz et al. [19] indicated that the production of CH_4_ and CO_2_ from pig intestinal fermentation is positively correlated with growth stages and dietary fiber content, and the GHG emissions from pigs vary across different stages. Thus, this method requires detailed country-specific data on nutrient requirements, feed intake, and CH_4_ conversion rates for specific feed types, which are used to develop emission factors for different countries. According to Table 3, enteric fermentation is responsible for 3.1% to 9.6% of the overall GHG emissions of the pig production chain.

### 3.5. Manure Management

The release of CH_4_ from manure originates from the temporal succession of microbial processes. Initially, bacteria convert easily degradable substrates into volatile fatty acids, CO_2_ and H_2_. This extensive microbial activity increases the temperature of the manure and provides suitable conditions for methanogenic bacteria to convert acetate, CO_2_, and H_2_ into methane under a thermophilic environment. Yuan et al. [24] reported that the carbon emissions from pig manure during composting are much higher than those from other livestock and poultry manure. Additionally, factors such as composting materials, initial carbon-to-nitrogen ratio (C/N), moisture content, and ventilation rate can affect GHG emissions. According to the guidelines for National Greenhouse Gas Inventories [22], CH_4_ emissions from manure can be estimated based on the amount of excreted volatile solid or organic matter; the ultimate CH_4_ potential depends on the region of the world, the climate, the livestock categories, and the type of manure. A recent study estimates the CH_4_ emission rate from manure using machine learning algorithms, at around 18 g per day per pig [25]. According to Table 3, manure management ranges from 19.0% to 56.9% of the overall GHG emissions of the pork production chain.

### 3.6. Wastewater and Harmless Treatment

The first step in the wastewater treatment process in pig farms is filtration via the grid system, which intercepts and removes various suspended solids and floating particles. The energy consumed by this process results from the operation of the machine. The second step is the collection system, which balances the wastewater and adjusts the volume. The main source of energy consumption in this process is the mixer. The third step in wastewater treatment is the solid–liquid separation system, which mainly separates the solid feces from the manure through screen filtration and screw pressing to achieve dry–wet separation. The main energy consumption comes from the operation of the solid–liquid separator. In anaerobic system processes, anaerobic bacteria are utilized to ferment dissolved organic carbon (DOC) in the wastewater, hydrolyzing DOC and producing CH_4_ [26], thus removing organic matter from the wastewater. The next step is the aerobic treatment of the effluent to further degrade organic pollutants and provide denitrification and phosphorus removal and improve the quality of the effluent for standard discharge. The electricity consumed by water pumps, circulation pumps, and blowers in this process contributes to carbon emissions during treatment.

Composting is one of the methods used to treat solid waste generated during production. It involves centralized collection, pile composting, fermentation, sterilization, and deodorization to achieve a harmless treatment and produce organic fertilizer for resource utilization. During this process, gases such as CO_2_, N_2_O, and CH_4_ are generated [27]. Furthermore, GHG emissions from waste treatment in pig farms account for 56.92% of total pig emissions [6]. The CO_2_ is produced through microbial respiration and rapid decomposition of organic matter during the composting process. The generation of N_2_O is attributed to the processes of ammonium nitrogen nitrification and denitrification. Urea in the feces is converted into NH^4+^ through the action of proteases or ureases. Under aerobic conditions, nitrification occurs, converting NH_4_^+^ into nitrite (NO_2_^−^) and further oxidizing it to nitrate (NO_3_^−^) [26]. Denitrification is a microbially mediated process that occurs under anaerobic conditions, reducing NO_3_^−^ to NO_2_^−^, nitric monoxide (NO), N_2_O, and nitrogen gas (N_2_). The CH_4_ is generated through anaerobic fermentation by methanogenic bacteria in organic waste [26]. The guidelines for National Greenhouse Gas Inventories specify the process for calculating N_2_O emissions from manure management. This calculation involves multiplying the total N excretion from pigs within each specific manure management system by the corresponding emission factor [22].

Another common method for slurry treatment is through anaerobic fermentation bed composting [28]. Anaerobic fermentation bed composting involves collecting the feces from the farm and evenly spraying them onto the bedding material in the fermentation tank using a spraying device. Specialized high-temperature microbial strains are added, and a turning machine is used to mix the feces and bedding material thoroughly. Under the action of microorganisms, the organic matter in the feces, such as crude protein, crude fat, residual starch, and urea, is degraded or decomposed into sugars, amino acids, or fatty acids. Acid-producing bacteria utilize these small molecular substances to produce CO_2_, water, and humus, generating heat in the process. The temperature in the central fermentation layer can reach 60–65 °C. Methanogenic bacteria utilize the carbon from DOC and CO_2_ as an electron acceptor to convert the products into CH_4_. After composting, the moisture is evaporated through turning, and the remaining small amount of residue is transformed into organic fertilizer.

The use of composting as a carcass disposal method is one of the harmless treatment methods; it breaks down organic materials using microorganisms. Microorganisms consume oxygen and feed on the organic substrates to produce GHGs. The other method is incineration, which consumes a large amount of energy and produces GHGs and harmful substances such as N_2_O, sulfides, and dioxins, which easily cause secondary pollution. In addition, the farm household waste and kitchen waste are composted, while the remaining household waste is mostly incinerated on-site. During this process, the combustion of plastic products can produce toxic substances such as dioxins, benzopyrene, and sulfur oxides, as well as GHG emissions.

### 3.7. Biosecurity

The carbon footprint of biosecurity measures on a farm can vary depending on the specific practices [29]. The construction and maintenance of biosecurity infrastructure, such as fencing, gates, biosecurity zones, and animal housing, contribute to the carbon footprint owing to use and transportation of materials and energy used during construction. Furthermore, biosecurity measures involve the use of energy-consuming equipment such as ventilation systems, heating or cooling systems, lighting, and surveillance cameras. Biosecurity practices involve regular cleaning and disinfection of facilities, which require water and energy inputs [30]. The carbon footprint can be influenced by the energy sources used for cleaning processes. The type of energy source used (e.g., electricity, fossil fuels) and the efficiency of the equipment will influence the carbon footprint. Biosecurity protocols on farms often require the transportation of feed, personnel, animals, and supplies [29,31]. The type of vehicles used, their fuel efficiency, and distance traveled can contribute to carbon emissions. It is important to properly dispose of biosafety waste such as used personal protective equipment or contaminated materials, the handling and disposal methods chosen may impact the carbon footprint. Implementing waste reduction and recycling practices can help minimize emissions associated with waste management. On the other hand, biosecurity measures may involve the use of disinfectants, cleaning agents, or pest control chemicals [32]. The production, transportation, and disposal of these chemicals can contribute to the carbon footprint on a farm, but it is still difficult to quantify the associated emissions (Table 4).

### 3.8. Slaughtering and Processing

The carbon footprint of pig production in previous studies varied from 0.6 to 6.75 kg CO_2_e·kg^−1^ pig live weight [3,6,7,8]. The carbon emission of slaughter and processing should not be ignored, even if such data are limited. Slaughtering and processing facilities require energy for various processes, such as refrigeration, ventilation, heating, and equipment operation [33]. Furthermore, slaughtering and processing operations often require water for cleaning, sanitation, and processing. Slaughtering and processing operations also use energy for water heating, sanitation, and processing, and emissions result from water treatment and wastewater management. Furthermore, refrigeration and cold storage in these facilities consume electricity [34]. Liu et al. [35] reported that slaughtering and processing of pork in China consume 81.2 kWh energy and produce 22.08 kg CH_4_ emission per 1000 kg carcass weight. Employing energy-efficient refrigeration technologies and using low-energy consuming refrigerants can help reducing carbon emissions. It is important to note that the carbon emissions associated with the slaughterhouse operations and the subsequent processing of meat products and co-products can vary depending on factors such as energy sources, waste management practices, and processing techniques employed. Wei et al. [9] reported the carbon footprint of pork is 3.8 kg CO_2_e/kg, and the proportion of slaughtering is 7% in the pork production chain. According to the LCA Food Database, the different pork products have different environmental impacts (Table 5). One serving of pork cuts (1 kg) is equivalent to 2.25 to 4.52 kg CO_2_e. A recent study indicated careful consideration of carbon footprint data, since it is crucial for dietary guideline development in order to promote both improved health and emissions reduction [36].

## 4. Mitigation Strategies for Carbon Emission

### 4.1. Reducing Transportation Distance

It is important to note that the transportation of animals to the slaughterhouse and the delivery of processed products to markets contribute to carbon emissions. By optimizing transportation routes, utilizing fuel-efficient vehicles, and consolidating shipments, the carbon footprint associated with transportation can be reduced. Several studies have demonstrated that reducing transportation distances can effectively decrease 13% to 18% of GHG emissions at this stage [4,5]. When vehicles travel shorter distances, they consume less fuel or electricity, resulting in lower energy consumption and reduced emissions.

### 4.2. Mitigation in Feed Crop Production

During the cultivation stage, the application of nitrogen-containing fertilizers constitutes a crucial emission source influencing the loss of reactive nitrogen during the growth of feed crops. Therefore, reducing the use of synthetic nitrogen fertilizers, optimizing fertilizer management, and increasing the rotation of leguminous crops can effectively reduce GHG emissions. Different feedstock materials exhibit varying carbon footprints due to differences in cultivation practices for feed crop production. Optimizing nitrogen fertilizer application rates by using the best estimate for an economic optimum nitrogen rate, growing crops with lower nitrogen requirements, and ensuring accurate application, such as calibrating application machinery, can improve efficiency and reduce nitrogen fertilizer application. Through the optimization of agricultural management practices (precision fertilization and efficient irrigation management), the input of chemical fertilizers during the cultivation of forage crops is reduced, leading to increased crop yields and diminished losses of reactive nitrogen during this growth stage. As a result, GHG emissions during the cultivation phase can be lowered by 13% to 22% [38]. Leguminous crops can biologically fix nitrogen, and increasing the incorporation of legumes in cultivated lands can reduce the need for nitrogen fertilizer application, thereby decreasing CO_2_, N_2_O, and NH_3_ emissions associated with fertilizer manufacture, transport, and application [39,40]. Additionally, nitrification inhibitors can slow the microbial conversion of ammonium-N to nitrate-N (nitrification), reducing the risk of loss through leaching or denitrification and thus increasing the nitrogen-use efficiency of fertilizers [41]. Urea is a commonly used nitrogen fertilizer in agriculture, and its use can lead to GHG emissions, primarily due to the decomposition process of urea releasing NH_3_ and N_2_O. Research has shown that the use of nitrification inhibitors and urease inhibitors not only reduces NH_3_ and N_2_O emissions but also lowers the extent of nitrogen leaching to the surface and the groundwater [42]. Furthermore, studies have indicated that inhibitors such as dicyandiamide and N-(n-butyl) thiophosphorictriamide can significantly reduce N_2_O emissions when applied in combination with both ammonium nitrate and urea fertilizers, reducing emissions considerably [43]. Adopting sustainable farming practices can effectively mitigate carbon emissions during this phase. Research has indicated that adopting organic agriculture can enhance soil organic carbon content, reduce chemical usage, and safeguard agricultural ecosystems, thereby efficiently sequestering carbon and reducing emissions [44]. Establishing an ecosystem-friendly crop planting structure can contribute to carbon emission reduction. The impact of regional planting structures on carbon emissions was investigated by analyzing variations in carbon emissions among different crops. The findings suggest that an increase in the cultivation areas of rice, maize, and peanuts would elevate carbon emissions, while wheat, sorghum, soybeans, and vegetables would see lowered emissions with expanded cultivation areas [45]. Furthermore, researchers have calculated the environmental effects of various land-use strategies, revealing that adopting sustainable and intensive cultivation practices could result in a reduction of over 50% in GHG emissions [8].

### 4.3. Renewable Energy Applications

The carbon footprint can be influenced by the energy sources used, with fossil fuels contributing more emissions compared to renewable energy sources. It is possible to increase the use of renewable energy sources to reduce the carbon emissions from production. Using renewable energy reduces dependence on fossil fuels like coal, oil, and natural gas, which are the primary sources of carbon emissions. Among all renewable sources, solar energy has the highest compatibility with production activities, but solar-powered operations suffer from high initial costs [46]. Further reductions in the cost of sensors and control units for solar energy conversion can address this problem to a large extent. Additionally, proper insulation operation in the pig pens during the autumn and winter seasons can prevent heat loss, thus reducing the electricity and fossil fuel consumption associated with heating to some extent. Equipment upgrades and replacement with energy-saving devices can be considered based on the specific conditions of the farming site. It is advisable to consider replacing old equipment with energy-efficient heating equipment and utilizing geothermal or renewable energy sources for heating purposes.

### 4.4. Energy-Saving and High-Efficiency Feed Processing Technology

**Figure 2:**
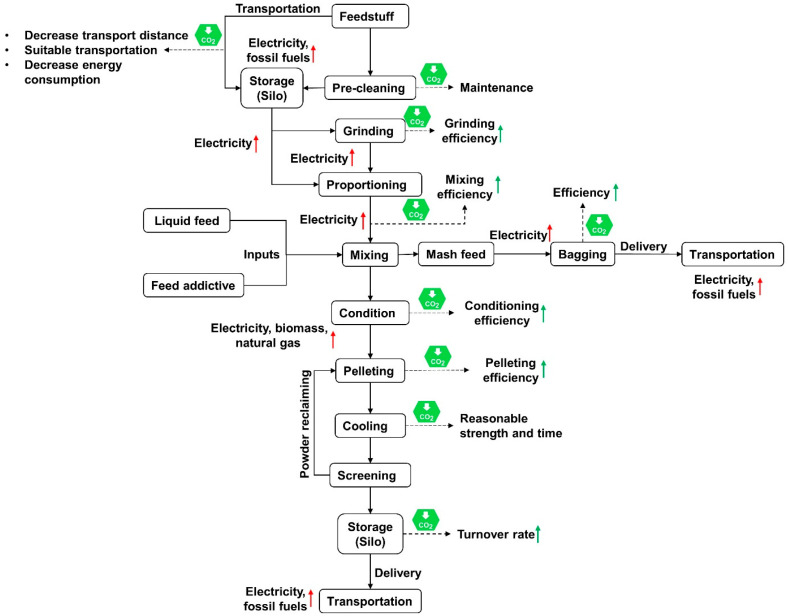
Schematic diagram of carbon emissions and mitigation in feed processing.

Based on the equipment and infrastructure conditions of the feed mill, it is necessary to optimize processing techniques. This aims to improve feed production efficiency and reduce the specific energy consumption of feed, thereby achieving carbon mitigation in feed production. In the grinding section, the feed frequency, negative pressure of the air damper, and clearance adjustment between the hammers and screens warrant timely monitoring and replacement of worn-out screens. Additionally, suitable particle sizes for material grinding should be matched according to the feed formulation design, thereby improving grinding efficiency and reducing electricity consumption. In a study conducted by Eras et al. [47], it was found that monitoring the wear degree of knives in hammermills through the temperature gradient of the feedstock during the milling process led to a remarkable decrease in electricity consumption by 32% and GHG emissions by 37% [47]. In feed production, the commonly used process is grinding before mixing (Figure 2), which requires multiple grinding bins and conveying equipment, resulting in high energy consumption. The process of mixing before grinding can be adopted to reduce the utilization of conveying equipment. However, it is important to consider the capacity of the grinding process and the full-load operation during material replacement. In the conditioning and pelleting process, the steam process should be adjusted according to the hydration of feedstuffs, suitable parameters for the ring die and pellet machine should be selected, and the working environment parameters should be matched to improve the yield and reduce the fine content of the final product. Regarding the prevention and control of African Swine Fever, pig feed production commonly involves high-temperature pelleting (above 85 °C) and increased conditioning time to ensure safety and reduce biological risks [48]. On the one hand, high temperature intake increases the energy consumption of the feed mill, and on the other hand, prolonged high-temperature processing may reduce the potency of active ingredients in the feed, such as vitamins [49]. Therefore, it is necessary to optimize the pig feed production process, determine the optimal processing parameters, and reduce energy consumption in the processing stage and at the same time ensure biological security.

### 4.5. New Modes of Environmental Control

Ventilation and heating are essential aspects of environmental control in pig houses. Ventilation plays a crucial role in regulating temperature, humidity, and oxygen levels, while simultaneously reducing the concentration of harmful gases, ultimately enhancing the overall comfort of the pig herd. To ensure sustainable production in pig farms, several recommendations can be considered. Firstly, the use of evaporative cooling terminals is suitable for dry areas, as proposed by Ignatkin et al. [50]. Secondly, a Chinese scholar introduced an innovative ventilation system combining a cooling pad and ground channel that can provide ample fresh and cooling air to meet the demands of large-scale pig farms [51]. To address the varying thermal comfort requirements of sows and piglets in the lactating house, the utilization of water-cooled pads can effectively resolve this issue through localized cooling [52]. However, it is important to consider the energy consumption associated with cooling operations when implementing these methods in farm facilities.

Insulation plays a crucial role in ensuring optimal environmental control within piglet houses. The vulnerability of newly born piglets to temperature fluctuations due to their underdeveloped bodies and limited thermoregulatory functions necessitates effective insulation measures. Traditionally, pig houses employ a combination of central heating plus localized heating lamps to provide heat. Central heating involves delivering hot air directly to the house through pipes using a hot air furnace. To minimize environmental impact, renewable energy sources can be considered as alternatives to coal or other fossil fuels, thereby reducing GHG emissions. Alternative heat sources, such as air-source heat pumps, can also be implemented, resulting in decreased CO_2_ emissions. Jeong et al. [53] reported that pig houses equipped with air-source heat pumps exhibited notable reductions in electricity consumption, total costs, and CO_2_ emissions. Moreover, enhancing the airtightness of pig house structures by incorporating environmentally friendly insulation materials on roofs, doors, and windows can further enhance thermal insulation and reduce heat transfer. These measures collectively contribute to energy-saving benefits and improved overall efficiency within pig houses.

### 4.6. Reasonable Manure Management

In medium-scale pig farming areas, the nearby farmland can deal with the manure. Using pig manure in nearby farmland as a substitute for a portion of chemical fertilizers can reduce CO_2_ emissions associated with fertilizer production [54]. Additionally, replacing energy-intensive chemical fertilizers with pig manure can decrease the overall energy demand for fertilizer production. Increasing the organic matter in the soil through pig manure application can enhance the carbon sequestration capacity of soil, thereby reducing atmospheric CO_2_ levels and mitigating global warming. However, it is important to note that nutrient content in pig manure varies among different farms due to variations in feed composition and pig digestion rates [55]. Proper composting practices, such as controlling the ventilation rate (0.1–0.3 L/kg/min), maintaining a moisture content of 60–65%, and achieving a C/N ratio of 20–25 [24], can effectively reduce GHG emissions. Before applying pig manure to the fields, it is necessary to determine the nutrient content of the manure and apply it to the nearby farmland in a manner that meets the nutrient requirements of the crops. It is recommended to regulate the scale of pig farming based on the capacity of land for manure assimilation and design pig feeding strategies based on the specific growth characteristics of local crops. During pig production, it is important to minimize the use of antibiotics on-site to prevent residual antibiotics from entering the environment and food chain, which can have adverse effects on human health [56].

Currently, pig production is characterized by intensification and large-scale operations, resulting in the production of manure exceeding the absorption capacity of nearby farmland. To address this issue, a combination of freezing and concentration technologies and membrane filtration can be employed to treat the manure. This involves anaerobic fermentation of the manure, processing the solid fraction into organic fertilizer through composting, treating the liquid fraction through reverse osmosis to meet the standards for wastewater discharge, and freezing and concentrating the remaining slurry to refine the remaining nutrients [57]. Large-scale pig farms should improve facilities for separating rainwater and wastewater, as well as implement water-saving measures in the pig house (such as regulating water flow and using water-saving drinkers) to reduce the overall volume of slurry. It is also advisable to install separate drainage pipes during the disinfection and cleaning of pens. Separate handling of wastewater can enhance the effectiveness of manure treatment. Furthermore, a significant number of trace elements (such as copper and zinc) are used in pig farming to meet the nutritional requirements of animals and promote growth, leading to their accumulation in pig manure. Research has shown that after pig manure is applied as fertilizer, the concentrations of copper, zinc, lead, and cadmium in the soil increase by 73%, 32%, 106%, and 127%, respectively [58]. The corresponding concentrations in the harvested brown rice from these soils increased by 104%, 98%, 275%, and 199%, respectively [58]. Given the large number of animals in large-scale farming operations and the accumulation of trace elements in pig intestines, it is necessary to regulate the use of additive products in pig feed to reduce the risk of heavy metal accumulation when applying manure to farmland.

### 4.7. Improve Pork Production Efficiency

Improving production efficiency is one effective approach to reducing carbon emissions. More efficient pig farming practices can lead to lower feed consumption, which in turn reduces the carbon footprint associated with production. By enhancing production performance, it is possible to reduce the number of pigs raised, thereby reducing GHG emissions during intensive farming. Improved pig production efficiency may require less land for raising feed crops or for the animals themselves. This reduction in land use can help preserve natural ecosystems and reduce carbon emissions associated with deforestation and land-use change. The following aspects can be considered for improvement:

- Adopting breeding goals: focused on increased feed efficiency and improved animal welfare that can lead to a reduction in the carbon footprint of pork production. The study compared two future scenarios, one reflecting the current breeding goal and the other reflecting a future breeding goal with the mentioned improvements; the results showed that the carbon footprint of the pork production system was 6.5% lower in the scenario with the alternative breeding goal compared to the one with the current breeding goal [59]. This suggests that by prioritizing feed efficiency and animal welfare, the pork industry can potentially produce pork with lower carbon emissions.
- Strengthen feeding management: Combined with the production model and local actual conditions, the standard operating procedures for pig production were formulated (establishing a stable workforce and deploy personnel according to production rhythm, implementing regulations on the use of farm hygiene products, strengthening biosecurity measures, and implementing effective management for the purification of epidemic diseases).
- Implement nutritional regulation methods: Adjusted nutrition based on the characteristics of different production stages to reduce carbon and nitrogen emissions in pig production. For example, the use of low-protein diets can reduce nitrogen and carbon content in pig manure, resulting in a significant reduction in GHG emissions during manure management processes [60,61]. The application of functional nutritional regulation techniques to improve feed conversion efficiency is key to reducing GHG emissions in the pig industry. Antimicrobial peptides, plant extracts, and functional substances can enhance the health and growth performance of pig herds [62,63,64,65], thereby strengthening immune function, reducing disease-related losses, and indirectly reducing the use of immunological healthcare products, thus lowering carbon emissions associated with their application.

## 5. Limitations of the Study

The pork production chain comprises several sectors. This study offers a preliminary overview of each sector. While the pork production chain involves various inputs, this study lacks carbon footprint data for these inputs. Because of the insufficient availability of relevant research, this study is unable to provide the specific carbon footprint of the pork cuts. Although current research offers numerous traditional single-emission reduction technologies, there remains a gap in evaluating the synergistic effects of emission reductions and a shortage of groundbreaking mitigation technologies. Although this article proposes specific emission reduction measures, the intricate and varied nature of agricultural production makes it challenging to predict the actual effectiveness of these mitigation measures. It is worth noting that this study, due to space limitations, may not cite all the contributions from scientists in this field.

## 6. Areas for Further Research

The future of carbon neutrality presents both challenges and opportunities, representing a significant transformation for economy and society. In the coming years, it will become imperative to address the following major technological challenges in alignment with the objectives of carbon neutrality: (1) Prioritizing source reduction, cleaner production processes, and high-quality recycling for reducing the carbon footprint of pork through digital ecological design. (2) Developing clean processes for waste reduction at the source. (3) Transforming waste materials into clean resources without generating further waste. (4) Implementing collaborative disposal methods for organic solid waste from various sources. (5) Smart disassembly and utilization of waste materials. (6) Controlling environmental health risks associated with chemicals. (7) Promoting pork industrial circular connections, covering core technologies, key materials, core components, software, intelligent equipment, and databases. 

## 7. Conclusions

Pork production is a significant contributor to carbon emissions in the agricultural sector, with key processes such as feed production, processing, and manure management being major sources of carbon emissions. The carbon footprint of pork production varies from 0.6 to 6.75 kg CO_2_e·kg^−1^ pig live weight, and the carbon footprint of 1 kg of pork products is equivalent to 2.25 to 4.52 kg CO_2_e. However, by adopting modern pork production technologies and implementing theoretical innovations, it is possible to achieve carbon mitigation throughout the entire pork production system. It shows a 6.5% to 50% reduction in carbon emissions in different stages. This transformation can lead to a shift from the high-energy consumption and heavy pollution status of pork production to a more sustainable and environmentally friendly approach characterized by low-carbon and net zero emissions. By addressing these factors and prioritizing carbon mitigation strategies, the pork industry can contribute to the broader goal of mitigating climate change and meeting future market expectations for environmentally sustainable industry.

## Figures and Tables

**Table 1:** The contribution of livestock species to global GHG emissions.

Species	GHG Emissions (Tg CO_2_ Equivalent/Year)			
	CO_2_	CH_4_	N_2_O	Total
Buffalo	89.29 (5.7%)	329.53 (8.9%)	94.53 (10.3%)	513.35 (8.3%)
Cattle	1029.35 (65.9%)	2300.46 (61.9%)	494.05 (54.0%)	3823.86 (61.8%)
Chicken	32.04 (2.1%)	424.86 (11.4%)	115.59 (12.6%)	572.49 (9.2%)
Goat	20.13 (1.3%)	173.07 (4.7%)	30.31 (3.3%)	223.50 (3.6%)
Pig	369.83 (23.7%)	325.09 (8.8%)	152.22 (16.7%)	847.13 (13.7%)
Sheep	22.02 (1.4%)	160.70 (4.3%)	27.44 (3.0%)	210.15 (3.4%)
Total	1562.66 (100%)	3713.70 (100%)	914.13 (100%)	6190.48 (100%)

Source: adapted from FAO [3].

**Table 2:** The GHG emission sources in the global pig industry.

Item	Percentage	Emissions (Megagram)
Direct on farm energy (CO_2_)	12.0	101,792,650.0
Embedded on farm energy (CO_2_)	8.2	69,774,277.0
Enteric fermentation (CO_2_)	33.2	281,072,770.7
Feed (CO_2_)	0.5	4,549,406.3
Feed (CH_4_)	6.0	50,422,302.7
Feed (N_2_O)	3.1	25,908,407.8
Land use (CO_2_)	3.2	26,893,778.6
Manure (CH_4_)	3.3	27,585,401.3
Manure (N_2_O)	28.5	241,017,553.3
Post farm (CO_2_)	2.1	18,104,037.9
Total	100.0	847,120,585.6

Source: FAO [3].

**Table 3:** The contributions of different sources to the carbon footprint of the pork production chain.

Reference	Transportation	Feed Production	Pig Production	Enteric Fermentation	Manure Management	Slaughtering
[2]	8.2%	42.6%	3.8%	3.1%	39.2%	-
[3]	-	46.0%	15.0%	5.0%	34.0%	-
[6]	2.2%	28.6%	2.7%	9.6%	56.9%	-
[7]	2.2%	27.8%	2.6%	9.3%	42.9%	-
[8] Smallholder	5.0%	26.0%	17.0%	-	50.0%	-
[8] Medium scale	10.0%	68.0%	11.0%	-	20.0%	-
[8] Industrial scale	10.0%	68.0%	14.0%	-	19.0%	-
[9]	-	65.0%	5.0%	4.0%	19.0%	7.0%

**Table 4:** The carbon footprint of biosecurity on farms.

Biosecurity Measure [29,31]	Carbon Footprint
Cleaning, disinfection, and drying of vehicles	Electricity, fuels, chemicals, and disinfectants
Quarantine, disinfection, and sampling to monitor pathogens of personnel entering	Disinfectant, reagents, and consumables
Disinfection, sampling, and testing of supplies	Electricity, disinfectants, reagents, and consumables
Quarantine for newly introduced pigs and sampling to monitor pathogens	Electricity, disinfectants, reagents, and consumables
Clean feed delivery truck	Electricity and fuels
Sick and dead pig treatment	Electricity, disinfectants, fuels, reagents, and consumables
Manure disposal and waste management	Electricity
Specific clothes and boots	Materials used and manufacturing processes
Full fencing around and closed farm area	Construction and maintenance processes
Strict control for entrance in farm	Disinfectants
Against birds	Materials used for the netting or mesh
Rodent control	Chemical usage

**Table 5:** The potential environmental impacts of pork products [37].

Impact Category	Unit	Pork 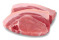	Tenderloin 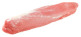	Ham 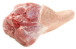	Minced Meat 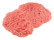
Global warming	kg CO_2_e	2.25	4.52	2.90	2.66
Acidification	g SO_2_e	40	75	48	28
Nutrient enrichment	g NO_3_e	214	413	266	207

## Data Availability

The data used to support the findings of this study can be made available by the corresponding author upon request.
